# Identification of a visualized web-based nomogram for overall survival prediction in patients with limited stage small cell lung cancer

**DOI:** 10.1038/s41598-023-41972-y

**Published:** 2023-09-11

**Authors:** Min Liang, Mafeng Chen, Shantanu Singh, Shivank Singh

**Affiliations:** 1 Department of Respiratory and Critical Care Medicine, Maoming People’s Hospital, Maoming, China; 2 Department of Otolaryngology, Maoming People’s Hospital, Maoming, China; 3 Division of Pulmonary, Critical Care and Sleep Medicine, Marshall University, Huntington, USA; 4 City Hospital, Shahjahanpur, India

**Keywords:** Cancer, Oncology, Risk factors

## Abstract

Small-cell lung cancer (SCLC) is an aggressive lung cancer subtype with an extremely poor prognosis. The 5-year survival rate for limited-stage (LS)-SCLC cancer is 10–13%, while the rate for extensive-stage SCLC cancer is only 1–2%. Given the crucial role of the tumor stage in the disease course, a well-constructed prognostic model is warranted for patients with LS-SCLC. The LS-SCLC patients' clinical data extracted from the Surveillance, Epidemiology, and End Results (SEER) database between 2000 and 2018 were reviewed. A multivariable Cox regression approach was utilized to identify and integrate significant prognostic factors. Bootstrap resampling was used to validate the model internally. The Area Under Curve (AUC) and calibration curve evaluated the model's performance. A total of 5463 LS-SCLC patients' clinical data was collected from the database. Eight clinical parameters were identified as significant prognostic factors for LS-SCLC patients' OS. The predictive model achieved satisfactory discrimination capacity, with 1-, 2-, and 3-year AUC values of 0.91, 0.88, and 0.87 in the training cohort; and 0.87, 0.87, and 0.85 in the validation cohort. The calibration curve showed a good agreement with actual observations in survival rate probability. Further, substantial differences between survival curves of the different risk groups stratified by prognostic scores were observed. The nomogram was then deployed into a website server for ease of access. This study developed a nomogram and a web-based predictor for predicting the overall survival of patients with LS-SCLC, which may help physicians make personalized clinical decisions and treatment strategies.

## Introduction

With morbidity of 2.26 million and mortality of 2.04 million in 2019, lung cancer continues to be an enormous health burden and leading the top cause of death worldwide^1^. Small-cell lung cancer (SCLC) accounts for up to 13–20% of lung malignancies^2^, characterized by rapid growth, early metastatic spread, and the most aggressive type of lung cancer with a poor prognosis. Despite a relatively high therapeutic efficacy upon initial treatment, most patients relapse owing to relative resistance, leading to adverse long-term outcomes. Over the last decades, very little progress has been achieved in SCLC patient survival in spite of the remarkable accumulation of knowledge regarding disease mechanisms^3^. Disease extent is one of the most critical prognostic factors contributing to SCLC patients' survival expectancy. It was studied that the median survival time among limited stage (LS)-SCLC patients range from 12 to 20 months, which is almost two times that of patients with extensive stage (ES)^4^. The American Joint Committee on Cancer (AJCC) and Veterans Administration Lung Study Group (VALSG) tumor staging systems are generally accepted as the most widely used predictive tool for SCLC patients in clinical practice. Unfortunately, outcomes can differ between the same stage tumors when applying the staging systems^5^. Furthermore, no conventional staging systems could use several normal clinical parameters responsible for the cancer prognosis^6–8^. Therefore, relying merely on traditional staging systems is not enough to accurately assess cancer prognosis in SCLC patients.

In light of the devastating prognosis and the crucial role of the tumor stage in the SCLC disease course, there is an urgent need to build a more precise and comprehensive model that will enable optimal therapeutic allocation and prognostication. It becomes crucial for LS-SCLC patients, given they are likely to derive more benefits from such interventions^9^. In recent years, predictive models, encompassing both machine learning and traditional methods like COX and logistic regression, have been gaining growing significance in the fields of molecular biology and clinical medicine^10,11^. Nomograms, as visual representations of prediction models, have gained widespread recognition as effective tools for prognosticating cancer patients. Their application in predicting patient outcomes can significantly contribute to formulating well-informed and personalized treatment strategies. Compared with the Tumor Node Metastasis (TNM) and the VALSG staging systems, nomograms outperformed in deriving more precise risk predictions and model visualization. Several nomogram studies are available on SCLC^8,12,13^. However, the studies included all staged SCLC patients and did not analyze patients in the limited stage particularly. It is a matter of concern because therapeutic strategies are varied in the two distinct populations. On the other hand, the existing models still have some shortcomings in the utilization of classification information and prediction performance.

Thus, in this study, with the data extracted from the Surveillance, Epidemiology, and End Results (SEER) database, we sought to establish a nomogram to assess the survival probability at 1-, 2-, and 3-year intervals in LS-SCLC patients. Furthermore, we compared the nomogram results with the TNM staging model developed in parallel to verify the model performance. Finally, a visualized web-based nomogram was established for its usability and visualized purpose.

## Methods

The ethics committee of Maoming People's Hospital approved the study protocol. Informed consent was not required because the SEER database does not contain personal information. In this study, model reporting complies with the Transparent Reporting of a Multivariable Prediction Model for Individual Prognosis or Diagnosis (TRIPOD) reporting guideline^14^. The methodology of model development and validation was partly adopted from a previous study^15^.

### Patient and data selection

This retrospective cohort study was based on a large population derived from the SEER database (SEER, https://seer.cancer.gov). The database was established by the department of cancer control and population sciences of the National Cancer Institute (NCI), which is an authoritative source of information on cancer incidence and survival in the United States. Therefore, the database has a good representation of clinicopathology, tumor features, and therapeutic details. In this study, the inclusion criteria included those patients who were pathologically confirmed LS-SCLC between 2000 and 2018. Exclusion criteria were as follows: incomplete demographic information such as age, sex, ethnicity, and marital status; incomplete clinicopathology information such as tumor size (defined as the most accurate measurement of a solid primary tumor in millimeter), tumor laterality, degree of tumor differentiation, TNM stage; incomplete therapeutic information such as surgery of the primary tumor site, chemotherapy, and radiotherapy; missing information regarding survival status and follow-up. According to the National Comprehensive Cancer Network (NCCN) and VALSG combined approach for SCLC staging^16^, LS-SCLC is defined as stage I to III (T any, N any, M0) in this study.

All primary data in this retrospective analysis was extracted from the SEER database with SEER ∗ Stata Software (version 8.3.9; https://seer.cancer.gov/data-software/).

### Statistical analysis

The primary endpoint was the overall survival (OS), defined as the interval from cancer diagnosis to the date of death reported in the registry. For clinical and demographic characteristics presented at baseline, frequencies and percentages were calculated for categorical variables, and mean and standard deviations were calculated for continuous variables. We randomly split the eligible patients into a training cohort and the remaining into a validation cohort in a 7:3 ratio. In addition to establishing the prediction model, the training cohort data was used to construct a nomogram and a classification system for risk assessment. In contrast, the data obtained from the validation cohort helped validate the model built by the training cohort.

We used Cox proportional hazards model to determine the effects of multiple factors on a nomogram. Specifically, univariate cox analysis was applied to determine the parameters associated with OS. Variables with statistical significance in univariate analysis were included in multivariate cox regression analysis to determine independent risk factors. A novel nomogram was constructed to predict the 1-, 2- and 3-year overall survival among LS-SCLC patients based on these independent factors.

The performance of the model was evaluated by applying the receiver operating characteristic (ROC) curve, calibration curve, and decision curve analysis (DCA). The predictive accuracy of prognostic models was assessed by area under curve (AUC) values of ROC curves. A greater AUC value translates into a more accurate prognostication. The accuracy of the nomogram was evaluated through the utilization of a bootstrap validation method with 1000 resamples on both the training and validation sets. Calibration refers to a model's accuracy of predicted risk probabilities, indicating the extent to which expected and observed outcomes agree. In a perfectly calibrated curve, the predictions should fall on the diagonal 45° line of the calibration plot. Finally, to estimate the clinical utility of this model, DCA was performed by calculating the net benefits for a range of threshold probabilities.

Furthermore, a risk classification system was established according to the total scores of each SCLC patient in the training cohort by applying the nomogram to separate patients into two prognostic groups, the low and high risk groups. Kaplan–Meier (K-M) curves were plotted based on the median risk score from each data as a cutoff to compare the survival risk between high risk and low risk groups.

All tests were performed using the R software (version 4.0.2, https://www.r-project.org/) with a two-tailed test, and p < 0.05 was considered to rule out the statistical discrepancy. The following R packages were applied during the model development: “rms”, “foreign”, “caret”, “survivalROC”, and “regplot”. The “DynNom” R package was used for web-based dynamic nomogram construction.

## Results

### Patient characteristics

**Figure 1:**
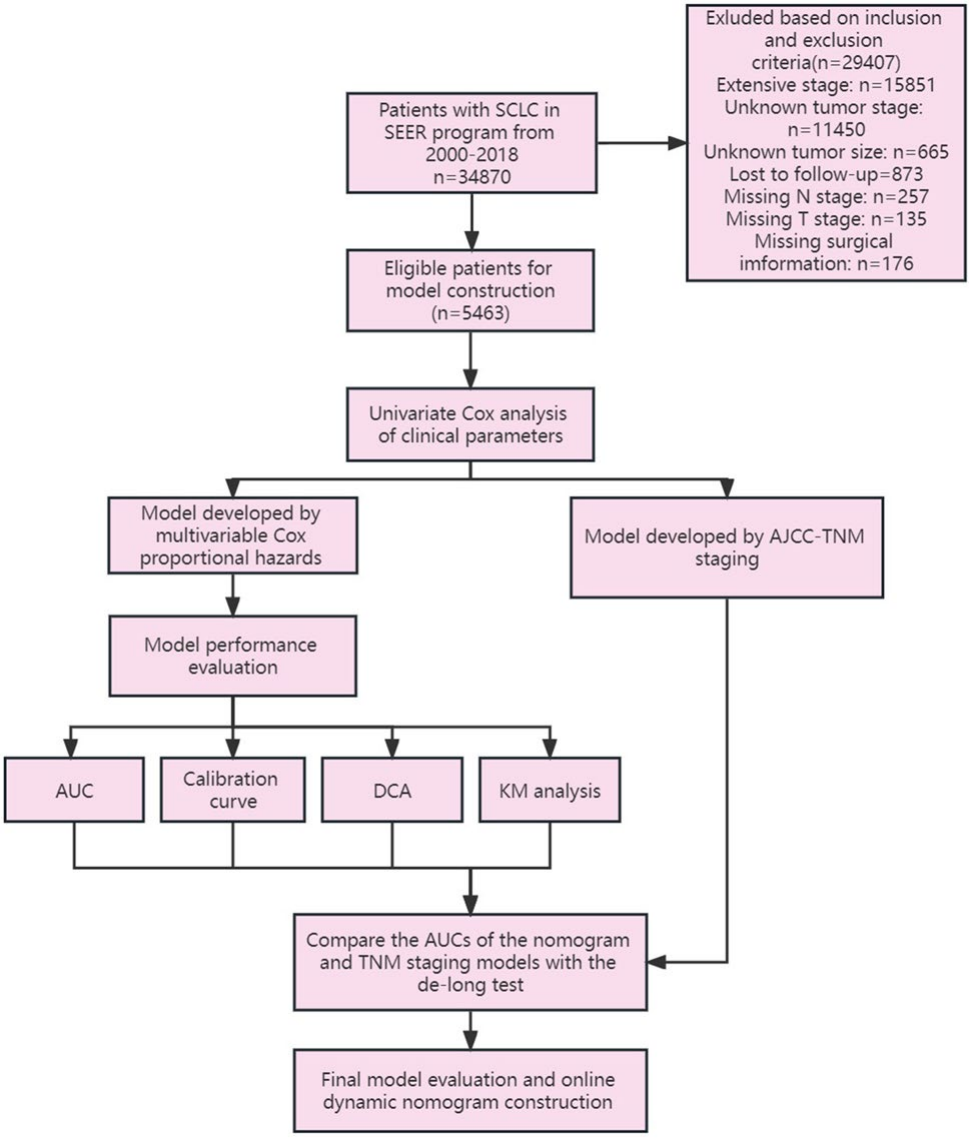
Flowchart of patient screening, enrollment, categorization, and model development.

Of 34,870 patients assessed for eligibility, 5463 patients met our inclusion criteria and were enrolled in the study. The screening process can be found in the flow chart (Fig. 1). With a median follow-up time of 15 months, 4622 deaths (84.6%) have been observed in the total population. Whites made up the majority of patients in the sample (85.7%), and the elderly consisted of over 95% of the population. The proportion of women was 9 percentage points higher compared to men. Married individuals constitute a slightly higher proportion compared to unmarried individuals.

Over 80% of patients have tumor sizes ranging from 3 to 7 cm, and most of the tumors occur in the right lung. For tumor differentiation, most patients exhibit tumors with a low degree of differentiation(poorly differentiated to undifferentiated).Concerning the therapy of SCLC, only a minority of patients were treated with surgery (9.6%). Over 80% of patients received chemotherapy, and the proportion of patients treated with radiotherapy was comparable to those who did not receive it.

Of the enrolled patients, 3824 and 1639 patients were randomly assigned to the training and validation cohorts for model construction and validation. The characteristics between the two cohorts were well balanced in terms of baseline patient demographics and clinical information (Table 1).

**Table 1:** Demographics, clinicopathologic characteristics, and treatment information of the enrolled LS-SCLC patients.

Variables	Description	Total population	Training cohort	Validation cohort	*p-*value
Number of patients		5463	3824	1639	
Age, n (%)					0.305
	≤ 40 years	22 (0.403)	20 (0.523)	2 (0.122)	
	41–50 years	227 (4.155)	157 (4.106)	70 (4.271)	
	51–60 years	1064 (19.476)	745 (19.482)	319 (19.463)	
	61–70 years	1957 (35.823)	1382 (36.140)	575 (35.082)	
	71–80 years	1641 (30.038)	1144 (29.916)	497 (30.323)	
	> 81 years	552 (10.104)	376 (9.833)	176 (10.738)	
Sex, n (%)					0.751
	Male	2461 (45.049)	1728 (45.188)	733 (44.722)	
	Female	3002 (54.951)	2096 (54.812)	906 (55.278)	
Marriage, n (%)					0.551
	Unmarried	2553 (46.733)	1793 (46.888)	760 (46.370)	
	Married	2682 (49.094)	1865 (48.771)	817 (49.847)	
	Unknown	228 (4.174)	166 (4.341)	62 (3.783)	
Race, n (%)					0.996
	White	4681 (85.686)	3277 (85.696)	1404 (85.662)	
	Black	544 (9.958)	380 (9.937)	164 (10.006)	
	Others	238 (4.357)	167 (4.367)	71 (4.332)	
Tumor size, n (%)					0.885
	≤ 3 cm	2090 (38.257)	1455 (38.049)	635 (38.743)	
	3.1–7 cm	2349 (42.998)	1651 (43.175)	698 (42.587)	
	> 7 cm	1024 (18.744)	718 (18.776)	306 (18.670)	
Laterality, n (%)					0.255
	Left	2225 (40.729)	1567 (40.978)	658 (40.146)	
	Right	3231 (59.143)	2254 (58.944)	977 (59.610)	
	Paired sites	7 (0.128)	3 (0.078)	4 (0.244)	
T stage, n (%)					0.545
	T1	1362 (24.931)	940 (24.582)	422 (25.747)	
	T2	1610 (29.471)	1134 (29.655)	476 (29.042)	
	T3	1082 (19.806)	773 (20.214)	309 (18.853)	
	T4	1409 (25.792)	977 (25.549)	432 (26.358)	
N stage, n (%)					0.425
	N0	1396 (25.554)	985 (25.758)	411 (25.076)	
	N1	587 (10.745)	398 (10.408)	189 (11.531)	
	N2	2727 (49.918)	1925 (50.340)	802 (48.932)	
	N3	753 (13.784)	516 (13.494)	237 (14.460)	
Grade, n (%)					0.371
	I (Well differentiated)	12 (0.220)	9 (0.235)	3 (0.183)	
	II (Moderately differentiated)	21 (0.384)	16 (0.418)	5 (0.305)	
	III (Poorly differentiated)	671 (12.283)	477 (12.474)	194 (11.836)	
	IV (Undifferentiated)	991 (18.140)	715 (18.698)	276 (16.840)	
	Unknown	3768 (68.973)	2607 (68.175)	1161 (70.836)	
Surgery, n (%)					0.983
	None	4939 (90.408)	3457 (90.403)	1482(90.421)	
	Yes	524 (9.592)	367 (9.597)	157 (9.579)	
Chemotherapy, n (%)					0.405
	No/unknown	1089 (19.934)	751 (19.639)	338 (20.622)	
	Yes	4374 (80.066)	3073 (80.361)	1301 (79.378)	
Radiotherapy, n (%)					0.933
	No/unknown	1801 (32.967)	1262 (33.002)	539 (32.886)	
	Yes	3662 (67.033)	2562 (66.998)	1100 (67.114)	
Status, n (%)					0.914
	Dead	841 (15.394)	590 (15.429)	251 (15.314)	
	Alive	4622 (84.606)	3234 (84.571)	1388 (84.686)	
Survival, median [IQR]	Reported in months	15.000[7.000,33.000]	15.000[7.000,33.000]	15.000[7.000,34.000]	0.679

*SCLC* Small cell lung cancer, *IQR* Interquartile range.

### Univariate and multivariate analyses

**Figure 2:**
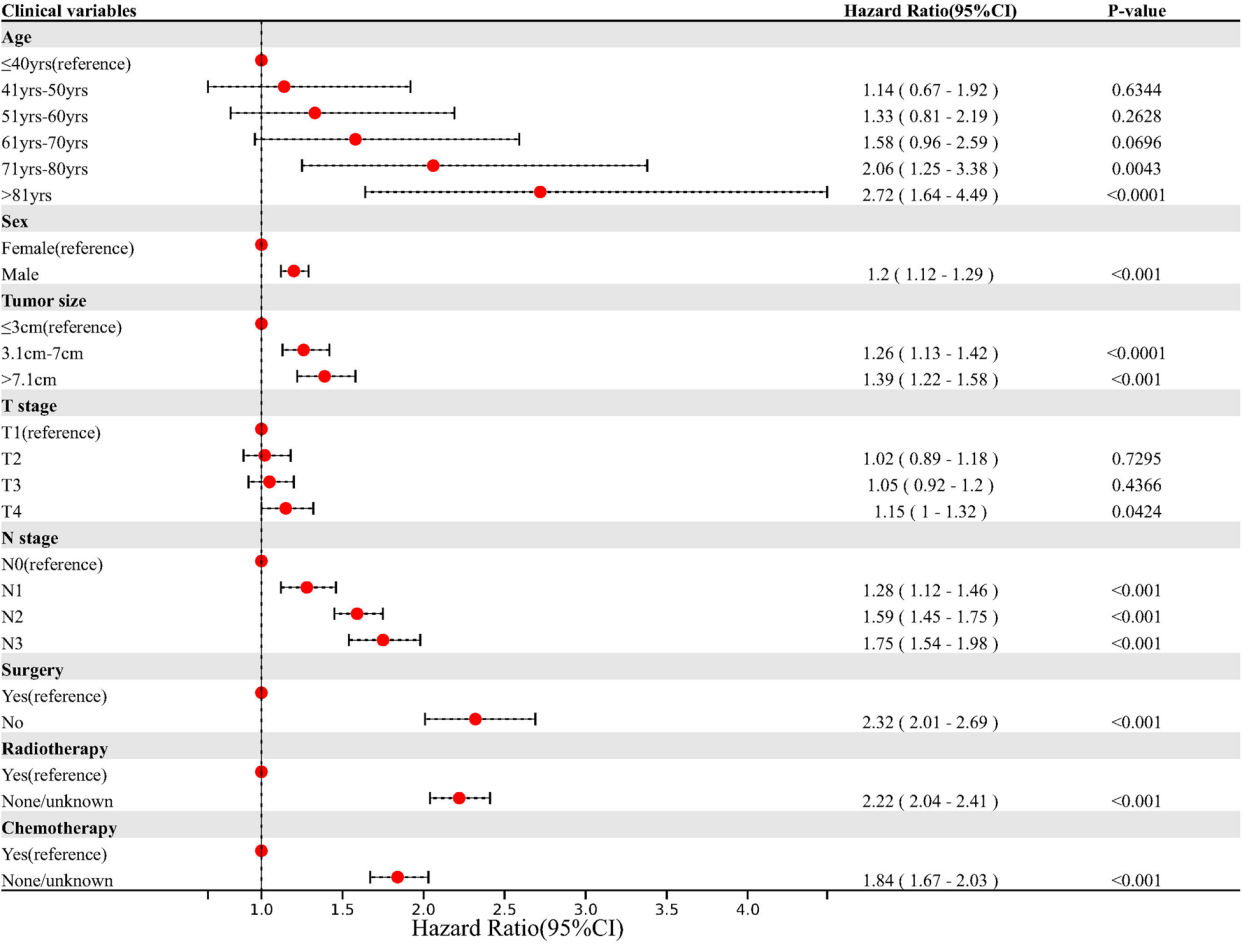
Forest plot of multivariate COX proportional hazards regression analysis to evaluate the prognostic factors for overall survival in limited stage small cell lung cancer patients.

The following parameters were entered into the Cox proportional hazards analysis: Age, gender, marital status, ethnicity, tumor size, tumor laterality, T stage, N stage, grade of tumor cell differentiation, surgery, chemotherapy, and radiotherapy. According to the results, all statistical significance factors with a p < 0.05 were entered into the multivariate Cox proportional hazards analysis. Age, sex, marital status, T stage, N stage, tumor size, surgery, radiotherapy, and chemotherapy were enrolled with a p < 0.001. The multivariate Cox proportional hazards analysis further revealed that age, sex, N stage, T stage, tumor size, surgery, radiotherapy, and chemotherapy were responsible for LS-SCLC patients' OS. The multivariate Cox proportional hazards analysis results can be found in Fig. 2.

### Prognostic nomogram development

**Figure 3:**
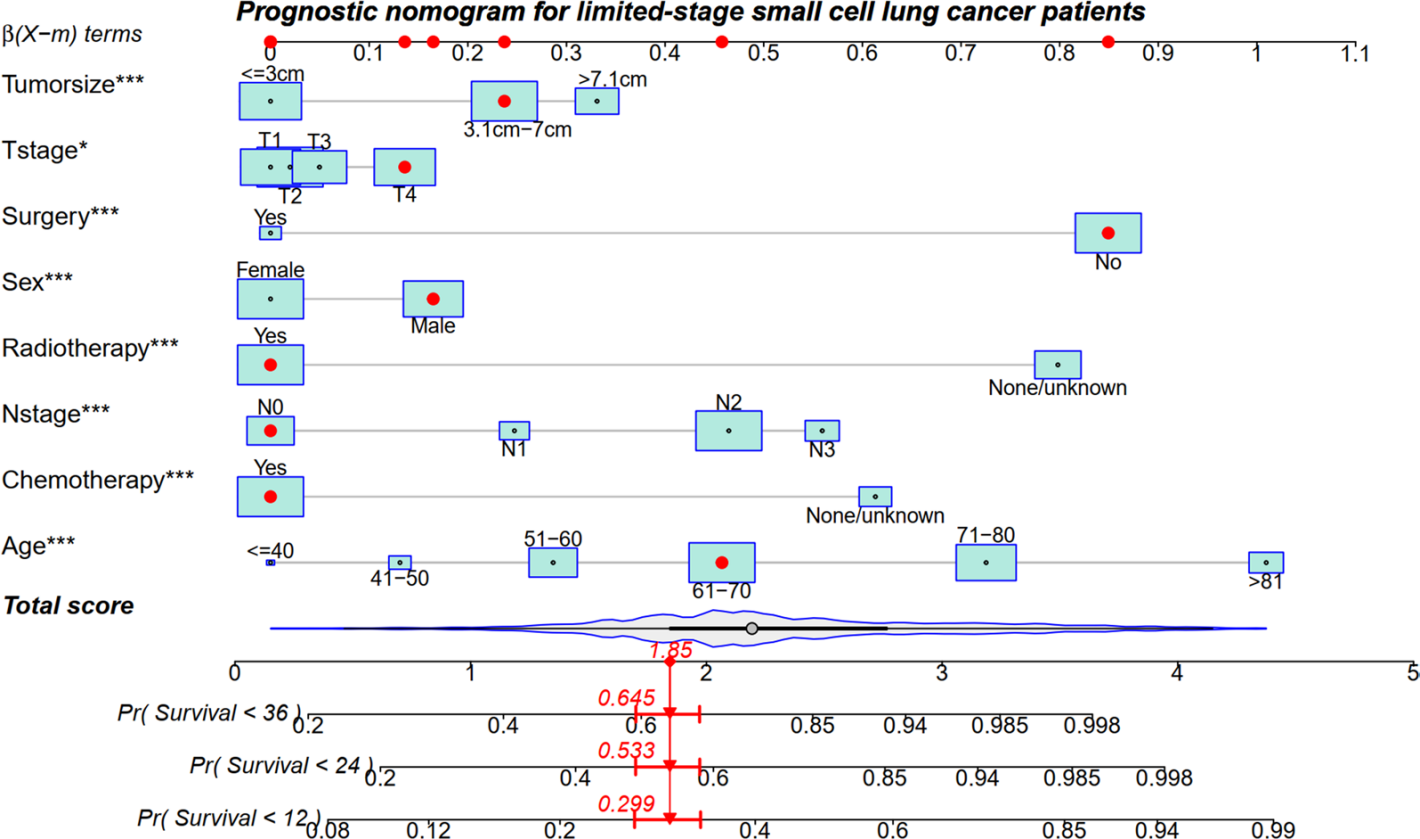
A nomogram for prediction of 1-, 2-, and 3-year overall survival for limited stage small cell lung cancer patients.

According to the result of the multivariate analyses, significant variables of age, gender, N stage, T stage, tumor size, surgery, radiotherapy, and chemotherapy were selected for nomogram construction. Each variable in the nomogram was assigned a point value from 0 to 1 based on ß coefficients in the multivariate model. The nomogram illustrated that age had the most considerable contribution to prognosis, with a point score of 1, followed by surgery and radiotherapy. An individual patient's risk score is calculated by adding the single points for each of the eight variables, and by adding the total score and finding where it falls on the survival scale, we can draw a straight line down to determine 1-, 2-, and 3-year survival probability. Higher scores among patients correlated with decreased survival. This provides clinicians and patients with a more informed understanding of the individual's prognosis, aiding in treatment decisions and discussions about potential outcomes(Fig. 3).

### Model performance and validation

**Figure 4:**
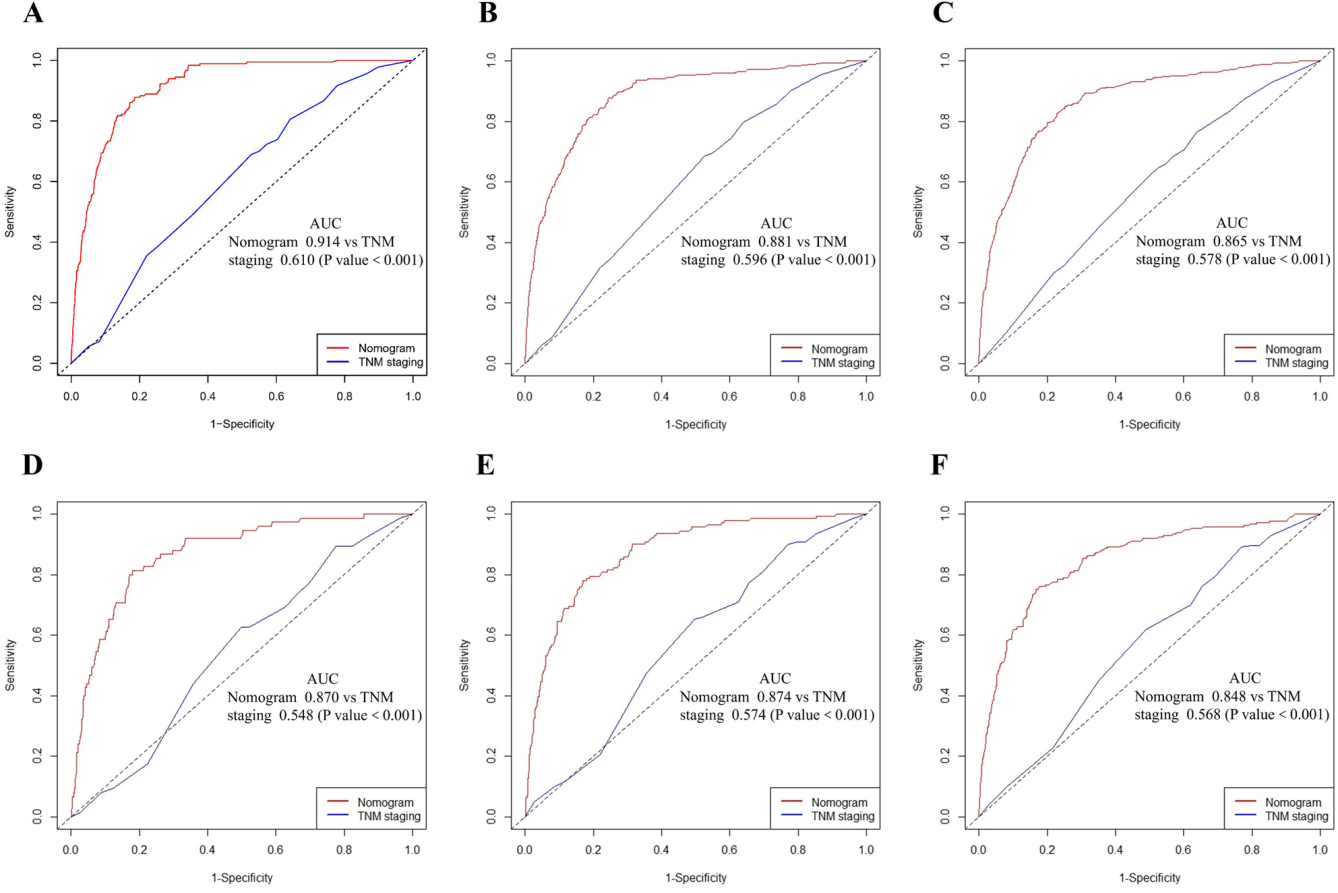
Comparison of nomogram and TNM staging for 1–2-, and 3-year overall survival prediction in limited-stage small-cell lung cancer patients: Receiver-operating characteristic curve (ROC) predict 1(**A**)-, 2(**B**)-, and 3(**C**)-year overall survival in the training set; ROC predicts 1(**D**)-, 2(**E**)-, and 3(**F**)-year overall survival in the validation set.

In the training cohort, the AUCs for the developed model were 0.91 (95% confidence interval [CI] 0.897–0.931), 0.88 (95% CI 0.863–0.901), and 0.87 (95% CI 0.848–0.883) for 1-,2-, and 3-year OS, respectively. While in the validation cohort, the AUCs for the constructed model were 0.87 (95% CI 0.831–0.909), 0.87 (95% CI 0.845–0.903), and 0.85 (95% CI 0.819–0.878) for 1-, 2-, and 3-year OS, respectively. To determine the predictive ability of our model, we also performed comparisons of the model AUCs between our nomogram and the TNM staging systems with the DeLong test. The 1-,2-, and 3-year time-dependent ROC curves of the two models can be found in Fig. 4. In the training cohort, AUCs predicting the nomogram's 1-,2-, and 3-year OS was significantly higher than the TNM staging system (p < 0.001). Similar results were obtained in the validation cohort compared to our nomogram with the TNM staging systems in predicting 1-,2-, and 3-year OS. Together, these results verified that our nomogram has a substantial prognostic value.

**Figure 5:**
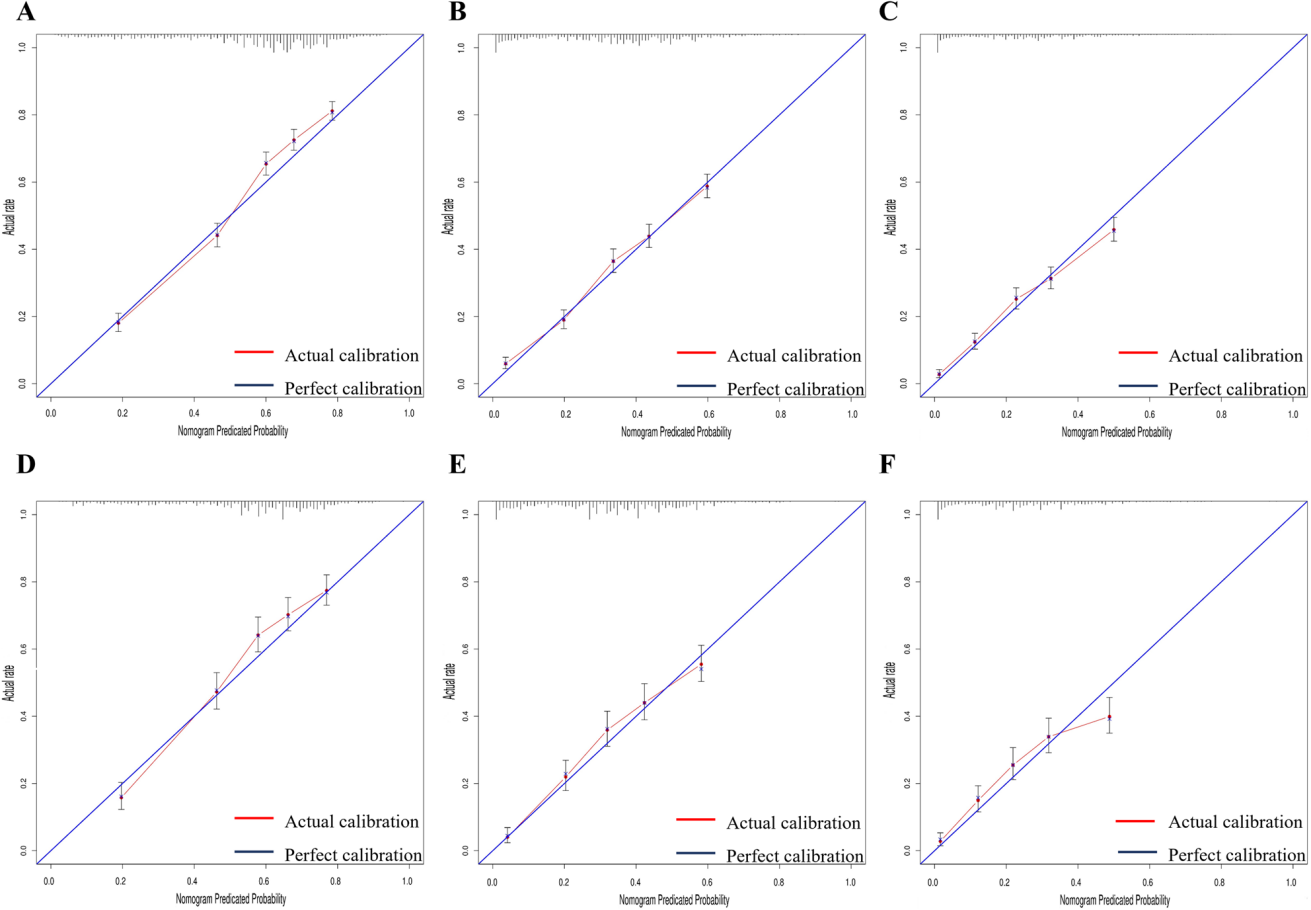
Calibration plots for nomogram-predicted overall survival (x-axis) and actual observed survival (y-axis) in limited-stage small-cell lung cancer patients: Calibration plots for 1(**A**)-, 2(**B**)-, and 3(**C**)-year overall survival in the training set. Calibration plots for 1(**D**)-, 2(**E**)-, and 3(**F**)-year overall survival in the validation set.

**Figure 6:**
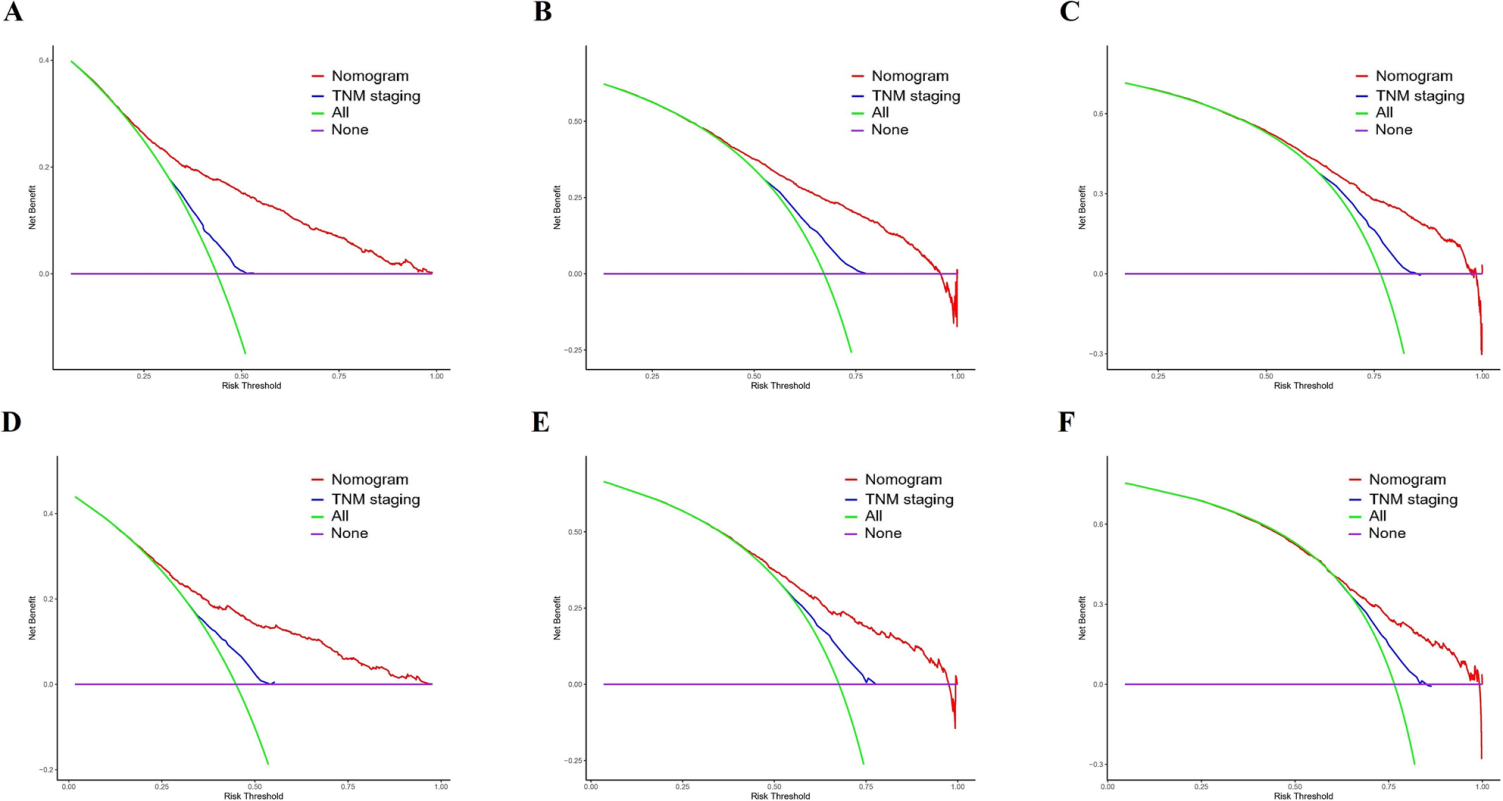
Decision curve analysis on the predictive model for limited-stage small-cell lung cancer patients: decision curve analysis for 1(**A**), 2(**B**), and 3(**C**)-year overall survival in the training set. Decision curve analysis for 1(**D**)-, 2(**E**)-, and 3(**F**)-year overall survival in the validation set. The x-axis represents the threshold probabilities, and the y-axis represents the net benefit.

Furthermore, as shown in Fig. 5, the calibration plots showed excellent consistency between the nomogram predictions and actual observations regarding the 1-, 2- and 3-year survival rates in the training and validation cohorts. In addition, the results of DCA also demonstrated that our nomogram has a high potential for clinical utility (Fig. 6).

### Development of the risk classification system

**Figure 7:**
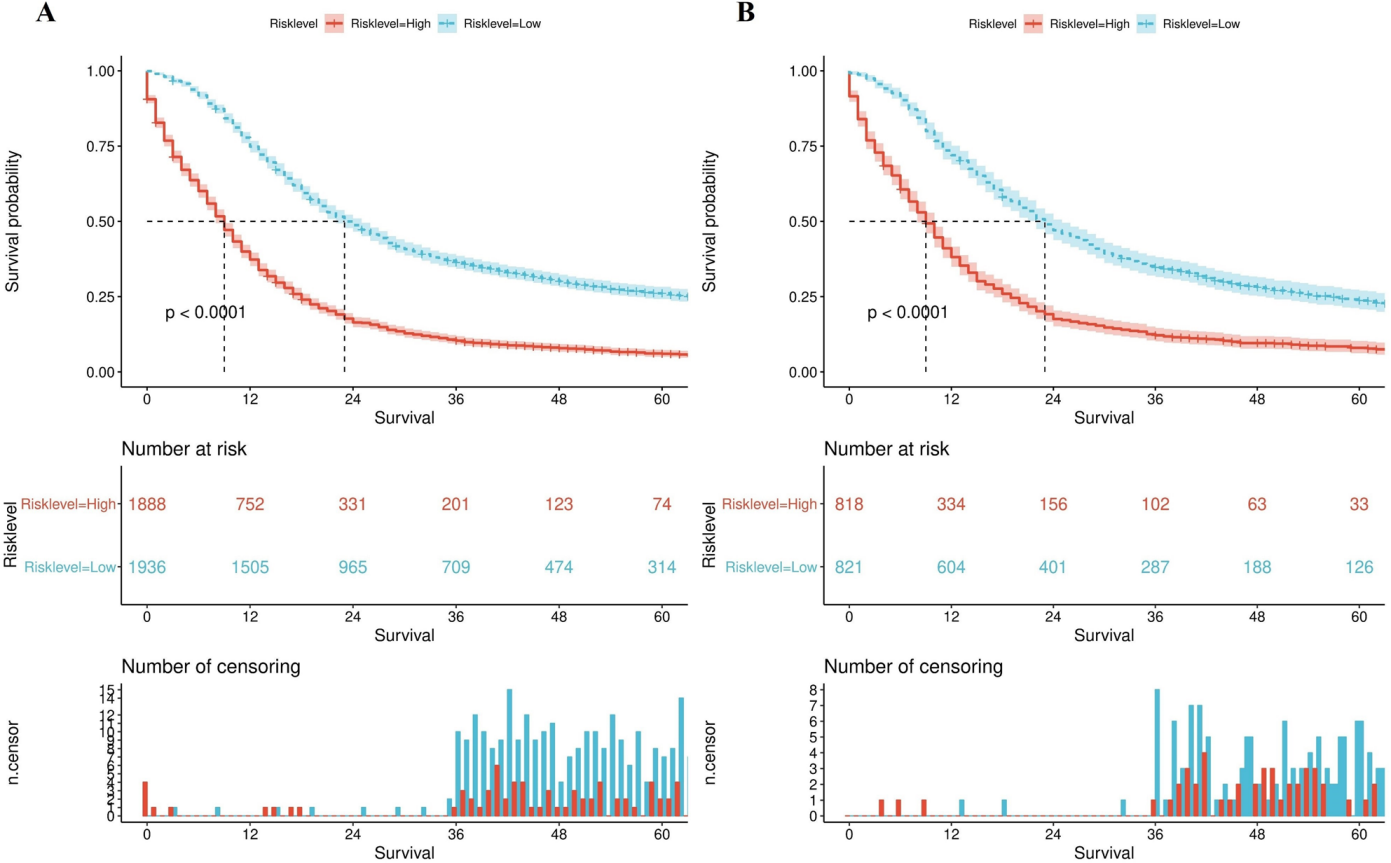
Kaplan–Meier curve analyses by the risk classification system for limited stage small cell lung cancer patients' overall survival in the training set (**A**) and validation set (**B**).

A predictive score model based on the nomogram in the training cohort was proposed to provide a quantitative tool for predicting risk classification. To describe the procedure in greater detail, we assigned the patients to high risk and low risk subgroups based on the cutoff value of the total risk scores. Detail subgroups were 8.98–79.44 for the high-risk population and 1.16–8.97 for the low-risk population. According to K-M curves, there is a clear difference between the two groups regarding survival. The log-rank test found significant differences between the two groups (p < 0.001). Similar results were also observed in the validation cohort when applicating the same grouping method (Fig. 7).

### Nomogram webserver development

**Figure 8:**
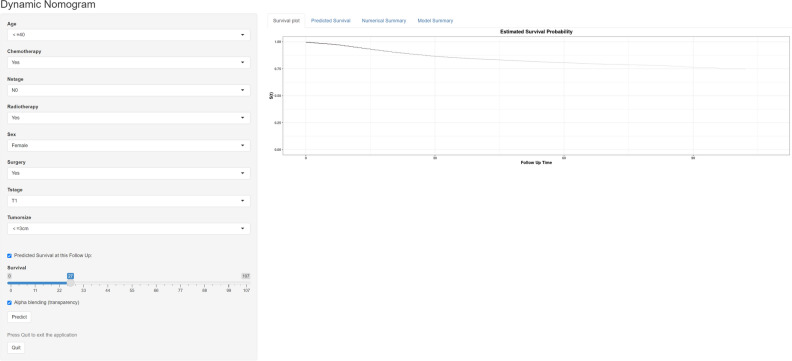
Online web server interface for the prognostic nomogram.

To support its application in clinical practice, we developed an online version of our nomogram based on a user-friendly website (Fig. 8). The development process relied on the identification of significant prognostic factors and obtaining coefficients for each predictor through univariate and multivariate regression analyses, utilizing the “DynNom” R package. Researchers and doctors can easily calculate the corresponding individualized predicted survival odds by plugging specific clinical data into the website (https://prognosticmodelforls-sclc.shinyapps.io/DynNomapp/).

## Discussion

Since SCLC remains a deadly disease with a therapeutic challenge and because of the crucial role of tumor stage in cancer prognosis, a well-developed prognostic model was warranted for LS-SCLC patients. In the present study, with a large sample of patient data derived from the SEER database, we developed and validated a prognostic nomogram to provide an individual survival prediction for LS-SCLC patients. Researchers and clinicians can quickly calculate the individualized probability of survival using clinicopathological variables and treatment information by utilizing our easy-to-use online calculator. Therefore, our study may facilitate clinical decision-making and assist in designing and interpreting future trials.

SCLC is known for its rapid growth and aggressive metastasis to multiple sites, along with a remarkable resistance to various therapies^17^. Patients with SCLC have not experienced significant benefits from advances in targeted therapies, and the improvements observed from the addition of immune-checkpoint inhibitor (ICI) therapy have been limited^18^. Therefore, the timely intervention in early-stage SCLC is essential to capitalize on the best treatment opportunities, maximize the chances of successful outcomes, and improve the overall quality of life for patients. It allows for a more aggressive and potentially curative approach to combat the disease before it progresses and becomes more challenging to treat effectively. Nowadays, a combination of TNM and VALSG classification systems approaches is the gold standard for SCLC patient prognostication. However, varying prognoses in patients may take place when applying these conventional tools. It is most likely due to the drawback that only a few variables are available to them. Given the biological individuality and complexity of the tumor, the traditional predictive methods are far from comprehensive. In recent years, increasing clinical parameters have been demonstrated to be associated with SCLC patient prognosis. With the application of such parameters, a more individualized treatment and prediction of survival could be achieved. The novelty of nomograms lies in its ability to integrate diverse patient characteristics, such as age, tumor stage, and biomarker levels, into a comprehensive predictive model. This innovative approach enhances precision in medical decision-making, enabling clinicians to tailor treatments and patients to gain valuable insights into their prognosis. The technicality of the nomogram stems from its statistical modeling, which involves rigorous data analysis, multivariate regression, and validation techniques to ensure accuracy and reliability^19,20^. As an interactive and visually appealing web-based application, the prognostic nomogram also empowers healthcare professionals and patients with a cutting-edge tool for improved risk assessment and informed healthcare choices.

Several nomograms have been developed to help consolidate and prognosticate SCLC patients' risk of death over time. For example, in 2017, using a single-institutional sample size of 450 patients as the training cohort, Xiao et al. constructed a prognostic nomogram for SCLC patients. The model achieved a predictive capability of a C-index of 0.60 among the population^12^. In another study conducted by Pan et al. in 2017^8^, a total of 275 SCLC patients were enrolled and used for the predictive model development. The C-index of their model was 0.68, compared to 0.65 in TNM staging. Regretfully, neither of the two models achieved a satisfactory predictive level, nor were the studies conducted based on a sufficient sample size of SCLC patients. In 2019, with a large sample size of 24,680 SCLC patient data collected from the National Cancer Database (NCDB), Wang et al. constructed a prognostic nomogram among this population. The model achieved a predictive power of AUC of 0.79^13^. Despite the large sample size, selection bias cannot be ruled out from the study because the authors incorporated the entire tumor stages and therapeutic strategies, including chemo-radiotherapy and surgery, which may generate bias from the interactions between tumor stages and therapeutic strategies. In clinical practice, the therapeutic schedule varies in tumor patients, most depending on the stage of disease and tolerability to the treatment^16^. Take SCLC as an example. More treatment options, either surgery or stereotactic body radiation therapy (SBRT), are available to LS-SCLC patients than those with ES-SCLC. In such a context, an appropriate predictive model with good performance is significant for reaching a reasonable treatment option and evaluating the prognosis in SCLC patients. On the other hand, the rapid growth of patients with SCLC has also highlighted the need for a more comprehensive and refined system for disease prognosis. Since our nomogram was specifically designed for LS-SCLC patients, such a model may provide more accurate survival probabilities for this subset of patients. It is supported by the results in the present study that the accuracy of our model was the highest relative to previous studies. In addition, our model was developed based on the vast geographically distributed database, which also ensures its generalizability for LS-SCLC patients.

Concerning the contribution of clinical parameters in LS-SCLC prognosis, variables sorted by the nomogram revealed that the most important independent prognostic factor was age, followed by surgery and radiotherapy. Moreover, sex, N stage, T stage, tumor size, and chemotherapy were also confirmed to be responsible for SCLC patient prognosis. These findings follow published studies and clinical guidelines^16,21–23^. However, it is interesting to note that chemotherapy is not among the top independent prognostic factors for SCLC survival. It is probably owed to the lack of neoadjuvant chemotherapy or postoperative adjuvant chemotherapy information in the SEER database. Sequentially, it further hampers us from investigating chemotherapeutic variants on cancer prognosis. It must, therefore, be acknowledged that the lack of such important information may result in an impact on the model's performance.

Regarding the reliability of our model, we applied model validation and calibration to prevent overfitting of the nomogram and verify its generalization in SCLC patients. The results are encouraging, as the calibration curves indicated outstanding agreement between the actual and model‐predicted survival probabilities, ensuring the validity of our model.

To further justify clinical utility, we performed DCA curves to assess the potential clinical effects of our model and obtained similar results. Based on the risk classification system, doctors can identify high-risk patients who may require additional treatment and intensive follow-up. However, direct use of the scoring system may not be appropriate, as multiple complex factors go into the doctor's decision to perform treatment, including personal and financial considerations, rather than merely the tumor stages.

Although this study has successfully established a prognostic model with good predictive power among LS-SCLC patients, it has limitations in study design, data collection, model validation, and interpretation. First, selection bias could not be avoided because of the retrospective nature of our study design. Second, despite the SEER database being a large repository, it is limited to the information stored. For instance, clinical data, such as radiotherapeutic intensity and chemotherapy, was only defined as yes, no/unknown in the database. In addition, comorbidities and laboratory tests are also not routinely available in this database. Over the past few years, there has been a notable surge in the development of prognostic models that combine genetic/protein-level data with clinical parameters. These models have demonstrated their significant role in disease prognosis and personalized treatment at the biomolecular level^24,25^.Therefore, it is important to acknowledge that the implementation of the current model has certain limitations. One notable constraint is the lack of patient genetic or proteomic information available in the SEER database, which hinders the full utilization of these advanced prognostic approaches. Efforts to expand data availability and improve the integration of genetic and proteomic data will be crucial for enhancing the effectiveness and applicability of the model in clinical settings. Finally, although we applied the bootstrap resampling method to avoid overfitting the model, the model should be validated externally.

## Conclusions

Our study built a reliable and clinically practicable nomogram based on a representative database, which can facilitate physicians in identifying high survival risk patients who may require adequate treatment and intensive follow-up to improve prognosis.

## Data Availability

Surveillance, Epidemiology, and End Results (SEER) provides the datasets generated during and analyzed in the current study [https://seer.cancer.gov/data/].

## Abbreviations

SCLC: Small-cell lung cancer
LS: Limited-stage
AUC: Area Under Curve
SEER: Surveillance, Epidemiology, and End Results
AJCC: American Joint Committee on Cancer
VALSG: Veterans Administration Lung Study Group
NCI: National Cancer Institute
NCCN: National Comprehensive Cancer Network
NCD: National Cancer Database
ROC: Receiver operating characteristic
DCA: Decision curve analysis
OS: Overall survival

## References

[1] Global Burden of Disease Cancer C, Kocarnik JM, Compton K, Dean FE, Fu W, Gaw BL (2021). Cancer incidence, mortality, years of life lost, years lived with disability, and disability-adjusted life years for 29 cancer groups from 2010 to 2019: A systematic analysis for the Global Burden of Disease Study 2019. JAMA Oncol..

[2] Jemal A, Bray F, Center MM, Ferlay J, Ward E, Forman D (2011). Global cancer statistics. CA Cancer J. Clin..

[3] Govindan R, Page N, Morgensztern D, Read W, Tierney R, Vlahiotis A (2006). Changing epidemiology of small-cell lung cancer in the United States over the last 30 years: analysis of the surveillance, epidemiologic, and end results database. J. Clin. Oncol..

[4] Lally BE, Urbanic JJ, Blackstock AW, Miller AA, Perry MC (2007). Small cell lung cancer: have we made any progress over the last 25 years?. Oncologist.

[5] Kalemkerian GP (2016). Small cell lung cancer. Semin. Respir. Crit. Care Med..

[6] Kang MH, Go SI, Song HN, Lee A, Kim SH, Kang JH (2014). The prognostic impact of the neutrophil-to-lymphocyte ratio in patients with small-cell lung cancer. Br. J. Cancer.

[7] Zhou T, He X, Fang W, Zhan J, Hong S, Qin T (2016). Pretreatment albumin/globulin ratio predicts the prognosis for small-cell lung cancer. Medicine.

[8] Pan H, Shi X, Xiao D, He J, Zhang Y, Liang W (2017). Nomogram prediction for the survival of the patients with small cell lung cancer. J. Thorac. Dis..

[9] Dingemans AC, Früh M, Ardizzoni A, Besse B, Faivre-Finn C, Hendriks LE (2021). Small-cell lung cancer: ESMO clinical practice guidelines for diagnosis, treatment and follow-up(☆). Ann. Oncol..

[10] Wang T, Sun J, Zhao Q (2023). Investigating cardiotoxicity related with hERG channel blockers using molecular fingerprints and graph attention mechanism. Comput. Biol. Med..

[11] Wang W, Zhang L, Sun J, Zhao Q, Shuai J (2022). Predicting the potential human lncRNA-miRNA interactions based on graph convolution network with conditional random field. Brief Bioinform..

[12] Xiao HF, Zhang BH, Liao XZ, Yan SP, Zhu SL, Zhou F (2017). Development and validation of two prognostic nomograms for predicting survival in patients with non-small cell and small cell lung cancer. Oncotarget.

[13] Wang T, Lu R, Lai S, Schiller JH, Zhou FL, Ci B (2019). Development and Validation of a Nomogram Prognostic Model for Patients With Advanced Non-Small-Cell Lung Cancer. Cancer Inform.

[14] Collins GS, Reitsma JB, Altman DG, Moons KG (2015). Transparent Reporting of a multivariable prediction model for Individual Prognosis or Diagnosis (TRIPOD): the TRIPOD statement. Ann. Intern. Med..

[15] Liang M, Chen M, Singh S, Singh S, Zhou C (2023). A visualized dynamic prediction model for overall survival in patients diagnosed with brain metastases from lung squamous cell carcinoma. Clin. Respir. J..

[16] National Comprehensive Cancer Network website. National Comprehensive Cancer Network Guidelines for small cell lung cancer. Available at: https://www.nccn.org/professionals/physician_gls/pdf/sclc.pdf. Accessed 17 Jan 2021.

[17] Rudin CM, Brambilla E, Faivre-Finn C, Sage J (2021). Small-cell lung cancer. Nat. Rev. Dis. Primers.

[18] Horn L, Mansfield AS, Szczęsna A, Havel L, Krzakowski M, Hochmair MJ (2018). First-line atezolizumab plus chemotherapy in extensive-stage small-cell lung cancer. N. Engl. J. Med..

[19] Balachandran VP, Gonen M, Smith JJ, DeMatteo RP (2015). Nomograms in oncology: More than meets the eye. Lancet Oncol..

[20] Sun F, Sun J, Zhao Q (2022). A deep learning method for predicting metabolite-disease associations via graph neural network. Brief Bioinform..

[21] Postmus PE, Kerr KM, Oudkerk M, Senan S, Waller DA, Vansteenkiste J (2017). Early and locally advanced non-small-cell lung cancer (NSCLC): ESMO Clinical Practice Guidelines for diagnosis, treatment and follow-up. Ann. Oncol..

[22] Shan Q, Shi J, Wang X, Guo J, Han X, Wang Z (2021). A new nomogram and risk classification system for predicting survival in small cell lung cancer patients diagnosed with brain metastasis: a large population-based study. BMC Cancer.

[23] Zeng Q, Li J, Tan F, Sun N, Mao Y, Gao Y (2021). Development and validation of a nomogram prognostic model for resected limited-stage small cell lung cancer patients. Ann. Surg. Oncol..

[24] Li X, Zhang P, Yin Z, Xu F, Yang ZH, Jin J (2022). Caspase-1 and gasdermin D afford the optimal targets with distinct switching strategies in NLRP1b inflammasome-induced cell death. Research.

[25] Xu F, Miao D, Li W, Jin J, Liu Z, Shen C (2023). Specificity and competition of mRNAs dominate droplet pattern in protein phase separation. Phys. Rev. Res..

